# AnCo3, a New Member of the Emerging Family of Phage-Like Plasmids

**DOI:** 10.1128/genomeA.00110-17

**Published:** 2017-05-11

**Authors:** Anna Colavecchio, Julie Jeukens, Luca Freschi, Jean-Guillaume Edmond Rheault, Irena Kukavica-Ibrulj, Roger Levesque, Lawrence Goodridge

**Affiliations:** a Department of Food Science and Agricultural Chemistry, Food Safety and Quality Program, McGill University, Ste-Anne-de-Bellevue, Canada; b Institut de Biologie Intégrative et des Systèmes (IBIS), Université Laval, Québec, Canada

## Abstract

A phage-like plasmid isolated from a clinical isolate of *Salmonella enterica* serovar Derby has strong nucleotide sequence identity to the phage-like plasmids pSTM_phi isolated from *Salmonella enterica* serovar Typhimurium L495, AnCo1 and AnCo2 from *Escherichia coli* 243 and *Escherichia coli* 244, and the virulent *Salmonella*-specific SSU5 bacteriophage.

## GENOME ANNOUNCEMENT

There are nine reported members of an emerging family of phage-like plasmids. They span different bacterial species, such as *Escherichia coli*, *Klebsiella pneumonaie*, *Yersinia pestis*, *Salmonella enterica* serovar Typhi, *Salmonella enterica* serovar Typhimurium, and *Acinetobacter baumanii* (1–7). These phage-like plasmids exist extrachromosomally and are characterized by double-stranded DNA genomes ranging from approximately 106 kb to 122 kb that contain structural bacteriophage genes as well as plasmid DNA.

A clinical isolate of *Salmonella enterica* serovar Derby was found to contain a phage-like plasmid. We performed whole-genome sequencing of the *S*. Derby isolate to determine the nature of the phage-like plasmid.

Whole-genome sequencing was performed at the EcoGenomics analysis platform (Ibis; Université Laval, Québec, Canada) on an Illumina MiSeq platform with 300-bp paired-end libraries and 30× coverage. The raw reads were assembled using the A5 pipeline (8). PHASTER (9) and PhiSpy (10) identified prophage regions. RAST (11) annotated the phage-like plasmid, while HostPhinder1.1 (12) and tRNAscan (13) identified the host range of the phage-like plasmid and tRNA genes, respectively.

The complete sequence of the phage-like plasmid from *S*. Derby, designated AnCo3, is 105,994 bp and has a G+C content of 46.4%. It also harbors 130 coding sequences (CDSs) and a tRNA for asparagine. Its host range is suggested to include *S. enterica* and *E. coli*.

Genomic analysis revealed that AnCo3 has strong nucleotide sequence identity to phage-like plasmid pSTM_phi (accession no. KP763470.1). AnCo3 shares 114 CDSs of 130 CDSs with pSTM_Phi, of which 96 CDSs have >95% nucleotide sequence identity and 18 CDSs have >75% nucleotide sequence identity. The bacteriophage genes of AnCo3 have >90% nucleotide sequence identity to pSTM_Phi, with the exception of a phage tail fiber with 71% nucleotide sequence identity. AnCo3 phage genes include an integrase, major capsid protein, tail fiber proteins, tail proteins, tail assembly proteins, a phage portal protein, the large subunit of the terminase, a holin, and an endolysin gene.

AnCo3 also has nucleotide sequence identity to the virulent *Salmonella-*specific bacteriophage SSU5 (GenBank accession no. JQ965645). In total, AnCo3 shares 97 CDSs out of 130 CDSs to SSU5 with >67% nucleotide sequence identity. The bacteriophage genes of AnCo3 have >73% nucleotide sequence identity to those of SSU5.

Nucleotide sequence identity was also detected to phage-like plasmids that infect *E. coli*, AnCo1 (accession no. KY515224) and AnCo2 (accession no. KY515225). AnCo3 shares 84 CDSs of 134 CDSs and 132 CDSs to AnCo1 and AnCo2, respectively, with >68% nucleotide sequence identity.

In conclusion, we present a novel phage-like plasmid isolated from a clinical *S*. Derby isolate. This is the 10th documented report of a phage-like plasmid; hence, we suggest that phage-like plasmids may represent a new genus of phages.

### Accession number(s).

The complete genome sequence of AnCo3 has been deposited in GenBank under the accession number KY515226.
